# The Post-Graduate of Prosthetic Dentistry

**Published:** 1889-11

**Authors:** 


### The Post Graduate School of Prosthetic Dentistry.

During a brief sojourn in Chicago recently, we had the pleasure of visiting the above named school and were exceedingly gratified to note its success and the practical methods of instruction which are followed in imparting knowledge to its pupils. The first thing taught is system and order, instruments and all apparatus must be in good condition for the uses they are to be put to. The laboratory room is furnished with all modern appliances of utility; the lathes are run by electric motor; furnaces for continuous gum work are in place, and facilities for every other kind of work used by the dentist is at hand so that the student upon entering wastes no time in preliminaries, but is immediately put to work, materials and tools in hand. The president of the institution, Dr. Haskell, is known throughout the dental world as one of the ablest mechanical operators in the profession. His ability to impart instruction is no less than his ability to perform, and his heart is so in the work of this school and his personal supervision so earnest, that no one possessing ordinary talent but what can bring themselves to a considerable degree of perfection under his teaching. There are other advantages of vast benefit to be derived from a course in this school. In the museum is a collection of hundreds of casts upon which practical dentures have been made, showing every variety of deviation imaginable from the normal mouth, the difficulties of successful adaptation, and the methods of overcoming them. In connection with the school, cases are received from dentists over the country which are made for them, thus assuring them of skillful and artistic dentures. This also furnishesthe student a vast opportunity for observation and comparison. While this school is mainly for the benefit of young men just entering the profession, yet it is available to any practitioner who may desire to brush the cobwebs of routine from before his eyes and learn the more recent methods of practice and manipulation. It is a lamentable fact, but a fact it is, that for the past twenty-five years, indeed, from the introduction of rubber as a material for the mounting of artificial teeth, mechanical or prosthetic (to use a little more aristocratic expression), dentistry has been neglected by the abler men of the profession until it has almost become a lost art, and those old masters of the art, who were not ashamed to the blush to be called mechanical dentists, have nearly all crossed over the ferry; but within the last few years, since "bridge" and "crown" have become the fashion, the profession seems to have taken a renewed interest in "prosthetic" dentistry and a revival has set in which may restore to it its past usefulness, and afford the toothless unfortunates an opportunity to secure dental substitutes that will beautify and naturalize their countenances, instead of changing their expression into that of the grinning skull. To this end such institutions as the one founded by Dr. Haskell and his colleagues, should be established at points where they might be sustained; especially where dental colleges are located, thus giving students additional advantages. W. T.

